# Cilostazol Prevents Endothelin-Induced Smooth Muscle Constriction and Proliferation

**DOI:** 10.1371/journal.pone.0044476

**Published:** 2012-09-05

**Authors:** Yoshifumi Kawanabe, Maki Takahashi, Xingjian Jin, Shakila Abdul-Majeed, Andromeda M. Nauli, Youssef Sari, Surya M. Nauli

**Affiliations:** 1 Department of Pharmacology, The University of Toledo, Toledo, Ohio, United States of America; 2 Department of Health Sciences, East Tennessee State University, Johnson City, Tennessee, United States of America; Medical University Innsbruck, Austria

## Abstract

Cilostazol is a phosphodiesterase inhibitor that has been shown to inhibit platelet activation. Endothelin is known to be the most potent endogenous growth promoting and vasoactive peptide. In patients and animal models with stroke, the level of circulating endothelin increases and complicates the recovery progress contributed by vascular constriction (an immediate pathology) and vascular proliferation (a long-term pathology). However, the effects of cilostazol on endothelin have not been explored. To demonstrate the dual-antagonizing effects of cilostazol on vasoconstriction and cell proliferation induced by endothelin, we used primary culture of mouse vascular smooth muscle cells *in vitro*, mouse femoral artery *ex vivo*, and intracranial basilar artery *ex vivo*. We show that the dual-inhibition effects of cilostazol are mediated by blocking endothelin-induced extracellular calcium influx. Although cilostazol does not inhibit endothelin-induced intraorganellar calcium release, blockade of extracellular calcium influx is sufficient to blunt endothelin-induced vasoconstriction. We also show that cilostazol inhibits endothelin-induced cellular proliferation by blocking extracellular calcium influx. Inhibition of cAMP-dependent protein kinase (PKA) can block anti-proliferation activity of cilostazol, confirming the downstream role of PKA in cellular proliferation. To further demonstrate the selectivity of the dual-antagonizing effects of cilostazol, we used a different phosphodiesterase inhibitor. Interestingly, sildenafil inhibits endothelin-induced vasoconstriction but not cellular proliferation in smooth muscle cells. For the first time, we show selective dual-antagonizing effects of cilostazol on endothelin. We propose that cilostazol is an excellent candidate to treat endothelin-associated diseases, such as stroke.

## Introduction

Cilostazol is a selective inhibitor of type-3 phosphodiesterase (PDE), and it is currently used to treat peripheral vascular disease of intermittent claudication [1], [2]. Thus, the clinical benefit of cilostazol is primarily set on its mechanism of action in preventing activation or aggregation of platelets. In addition to its action on platelets, it has been shown that cilostazol can promote vasodilation [3]. Though the exact mechanism of this vasodilatory effect is still debatable, it is known that cilostazol can reduce cytosolic calcium concentration, which plays a key role in this vasodilatation [4]. Furthermore, cilostzol has been shown to decrease cellular proliferation [5], [6]. Whether or not this action on cellular proliferation depends on cytosolic calcium remains to be determined.

**Table 1:** Primer Sequences.

PDEs	Accession Numbers	Primer Sequences	Positions
PDE1	NM_001159582.1	Forward: 5′-TCCTGCCCTGCTTCAGCTGCTA-3′	119
		Reverse: 5′-GGCGCTGCCACATCTTCTCGG-3′	433
PDE2	NM_001143848.2	Forward: 5′-GCCACTCACTGCTGCCTCCTG-3′	991
		Reverse: 5′-CCTCATCTGTGAAGCTGGAAGTCGG-3′	1315
PDE3	NM_018779.1	Forward: 5′-CGACCCTTCTCTGCCCCCGA-3′	1398
		Reverse: 5′-GAAGGGGCTTCGTGCGGCTT-3′	1730
		Forward: 5′-TACCGCAGTGAGCGAGCGAG-3′	23
PDE4	NM_019798.5	Reverse: 5′-AGGAGGTTTGGCCCAGTCCCTG-3′	373
		Forward: 5′-TACAGCGGAGCGAGCACCCG-3′	2052
PDE5	NM_153422.2	Reverse: 5′-ATCGCAGAGAGGTCGCAGGC-3′	2361
		Forward: 5′-GGAAGAGATCGTCGGCGTGGC-3′	1288
PDE6	NM_146086.2	Reverse: 5′-CTCCGTGAGTGGCAGGTCGC-3′	1651
		Forward: 5′-AGTGCCCCCTCTCTTCTGCCC-3′	5
PDE7	NM_008802.2	Reverse: 5′-TGTTCCTGGCGGAGACTGATGCT-3′	334
		Forward: 5′-TGTCCCACCACGGATTGCCCT-3′	1545
PDE8	NM_008803.2	Reverse: 5′-CCCGCGTTGCACAGGAAGGA-3′	1931
		Forward: 5′-GGTGGCAGAACCGTGGGTGG-3′	1284
PDE9	NM_008804.4	Reverse: 5′-AGTCTGTGCACACATTGAGATGTCC-3′	1685
		Forward: 5′-CGCCGGTGGCCGAACTCTTT-3′	253
PDE10	NM_011866.2	Reverse: 5′-GAGCAGGTGGTTGTCCCCGC-3′	649
		Forward 5′-TGGTGGACGCTGACCGTTGC-3′	741
PDE11	NM_001081033.1	Reverse: 5′-TCGCCTGGGCCACACCGATA-3′	1070

Most importantly, the clinical benefits of cilostazol for its dual-actions on vasodilation and anti-proliferation have not been explored. We here propose that cilostazol has a unique property for endothelin-associated diseases, such as proliferative vasospasm. In particular, endothelin plays a major role after stroke and further contributes significantly to complications in morbidity and mortality. It has been known that after stroke, the level of circulating endothelin increases in both patients [7]–[9] and animal models [10], [11]. In smooth muscle cells, the immediate action of endothelin is to trigger vasoconstriction (vasospasm), and its long-term effect is to promote cellular proliferation [12], [13]. Thus, the present study aims to examine the anti-vasoconstrictory and anti-proliferatory effects of cilostazol in endothelin-induced models, such as stroke.

We show for the first time that the dual actions of cilostazol can prevent endothelin-induced vasoconstriction and cell proliferation in mouse smooth muscle cells *in vitro* and *ex vivo*. Cilostazol prevents endothelin-induced extracellular calcium influx but not intraorganellar calcium release. Furthermore, the anti-proliferation action of cilostazol depends on the activation of cAMP-dependent protein kinase (PKA) signaling pathway. Together, we propose that cilostazol may have a therapeutic benefit to patients with stroke where endothelin has been shown to complicate the post-stroke recovery process [7]–[9].

## Materials and Methods

All animal procedures were approved by The University of Toledo animal care and use committee. The use of primary cells and other biohazard reagents was approved by the Institutional Biosafety Committee of The University of Toledo. In our current studies, we used isolated primary smooth muscle cells from mouse femoral arteries *in vitro*. We also utilized freshly isolated mouse femoral and intracranial arteries *ex vivo*. The isolation of primary cells is routinely performed in our laboratory [14], [15], and primary cultures of smooth muscle cells were confirmed with flow cytometry and maintained at 39°C in Dulbecco’s Modification of Eagle’s Medium (DMEM) containing 15% fetal bovine serum (FBS).

**Figure 1:**
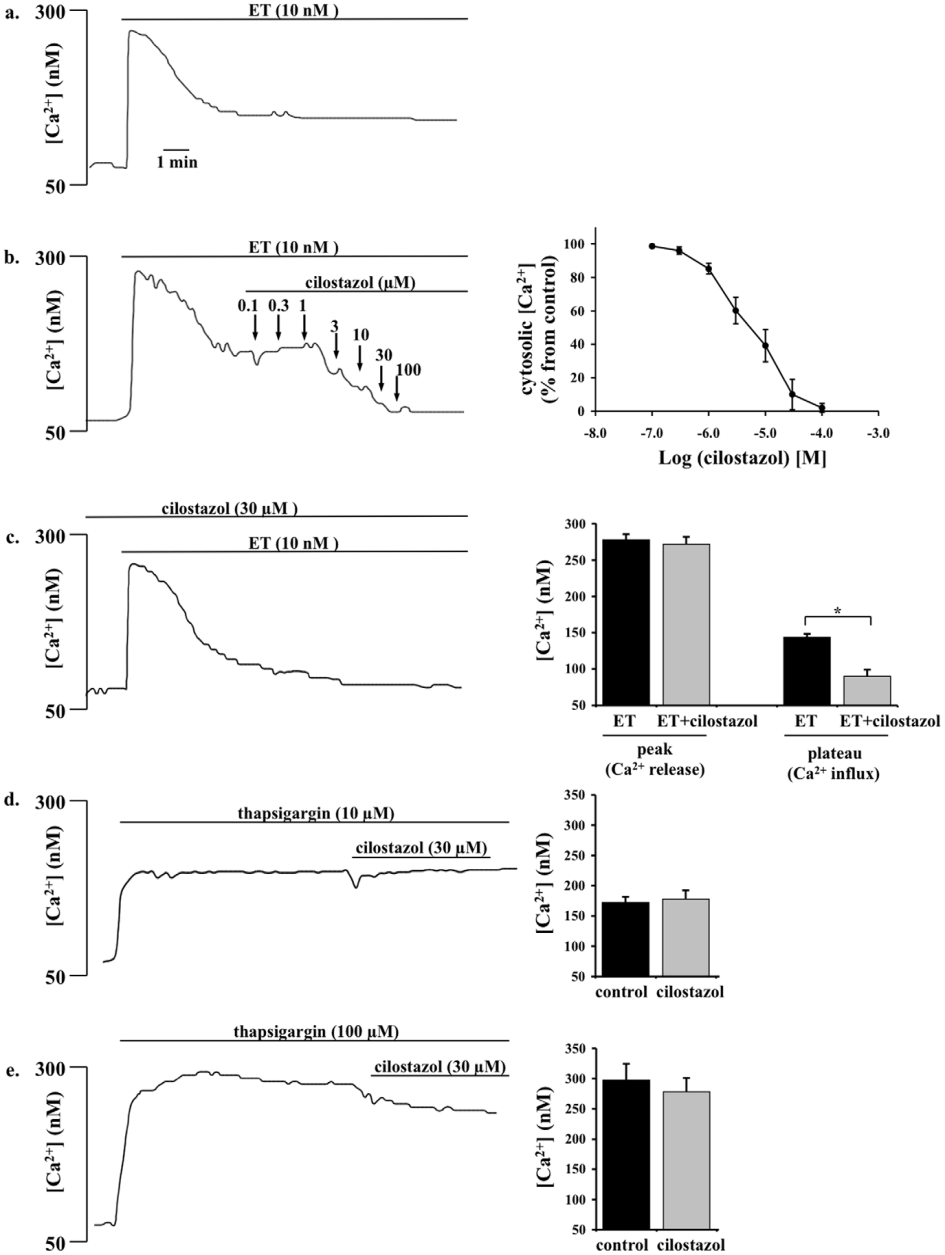
Cilostazol inhibits endothelin-induced extracellular calcium influx in a dose-dependent manner. a. Endothelin (ET) increases intracellular free calcium in primary culture of mouse femoral smooth muscle cells in a biphasic manner. ET induces intraorganellar calcium release (initial peak) and extracellular calcium influx (plateau phase). **b.** After the intracellular calcium increase is stabilized, dose-dependent effects of cilostazol is examined by applying various concentrations of cilostazol to the cells. Percent changes in cytosolic calcium is presented on the right panel (N = 4). **c.** Pretreatment with sub-optimal cilostazol concentration blocks endothelin-induced calcium influx but not calcium release. Effects of endothelin-induced calcium influx and release are quantified in the presence or absence of cilostazol pretreatment (N = 5). **d.** 10 µM of thapsigargin promotes release of intraorganellar calcium release. After the intracellular calcium increase is stabilized, sub-optimal concentration of cilostazol does not show a substantial effect on calcium release (N = 4). **e.** Cilostazol has no significant role in 100 µM thapsigargin-induced calcium release (N = 5).

### Cytosolic Calcium Measurement

Isolated femoral (∼200 µm in diameter) or basilar (∼50 µm in diameter) artery was cut into small pieces to form cylinder-like shapes. Artery segments or isolated primary smooth muscle cells were incubated with 5 µmol/L Fura2-AM for 30 minutes at 39°C as previously described [14], [15]. Basal calcium was equilibrated for about 5 minutes in Dulbecco’s Phosphate-Buffered Saline (DPBS). Paired fluorescence images of Fura2 at excitation wavelengths of 340 and 380 nm were recorded at every 4 seconds using Nikon TE2000 microscope and analyzed using Metafluor software.

**Figure 2:**
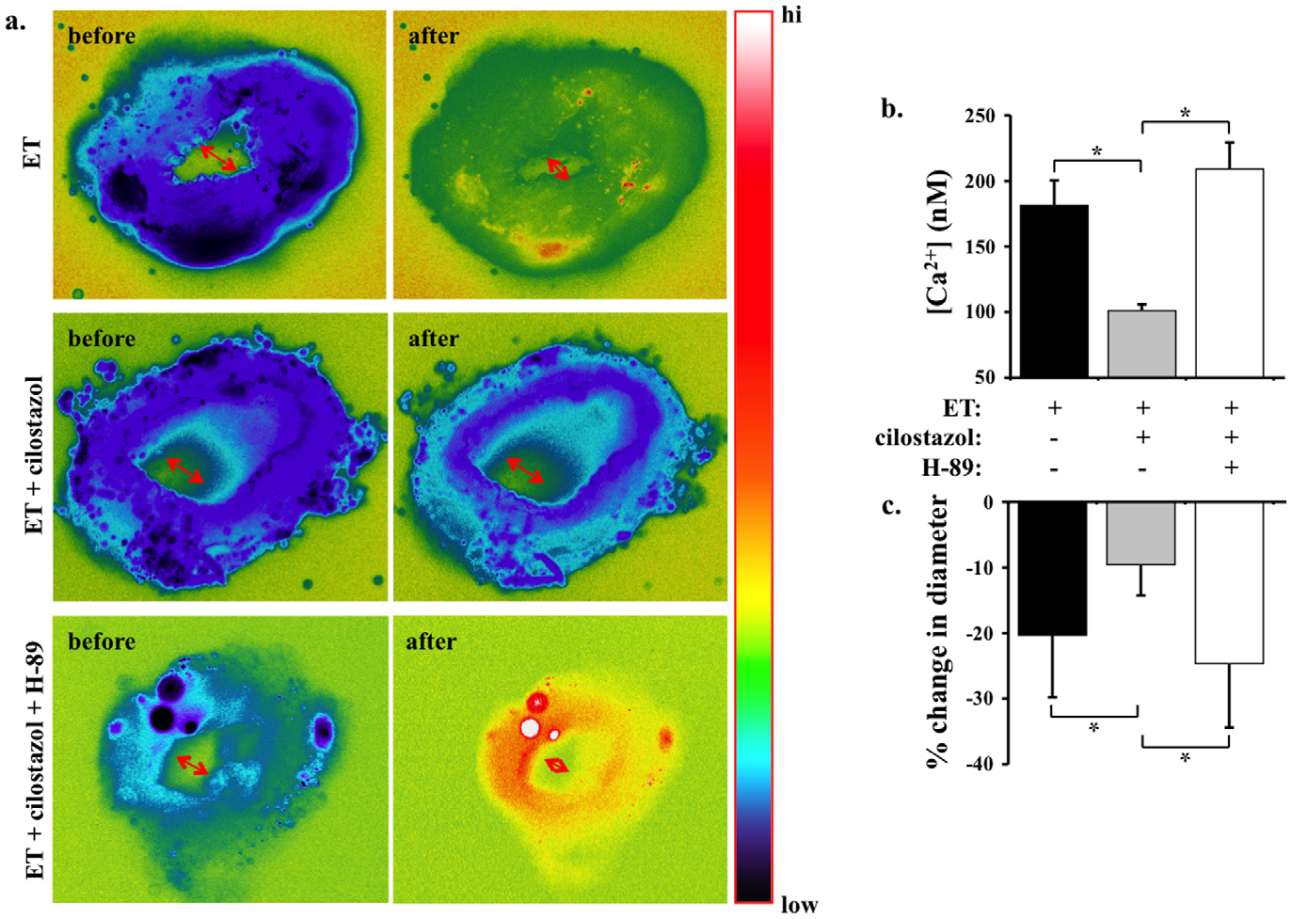
Cilostazol inhibits endothelin-induced cytosolic calcium increase and vasoconstiction in mouse femoral arteries. a. Cytosolic calcium (pseudocolor) and inner diameter (arrows) of femoral arteries are studied *ex vivo*. Segment of artery is imaged and recorded before and after endothelin (ET) treatment in the presence or absence of cilostazol and/or H-89. Artery segment is pseudocolored to denote changes in cytosolic free calcium level from low to high (hi) as indicated in the color bar. **b.** Cilostazol significantly decreases endothelin-induced cytosolic calcium increase. **c.** Changes in diameter denoting vascular constriction are expressed in percentage relative to baseline diameter prior to endothelin treatment. N = 5.

### Flow Cytometry Analysis

Primary smooth muscle cells were detached from femoral arteries with trypsin-EDTA. After growing in culture for about 1 week, cells are permeabilized with ice-cold 100% ethanol. Total RNA was digested with 0.5 mg/ml RNase A (*Boehringer Mannheim, Inc.*) at 37°C for 30 minutes. The cells were then incubated with 20 µg/ml propidium iodide (PI; *Sigma, Inc.*) for 1 hour at room temperature before analyzed with C6 Flow Cytometer (*Accuri Cytometers, Inc.*).

**Figure 3:**
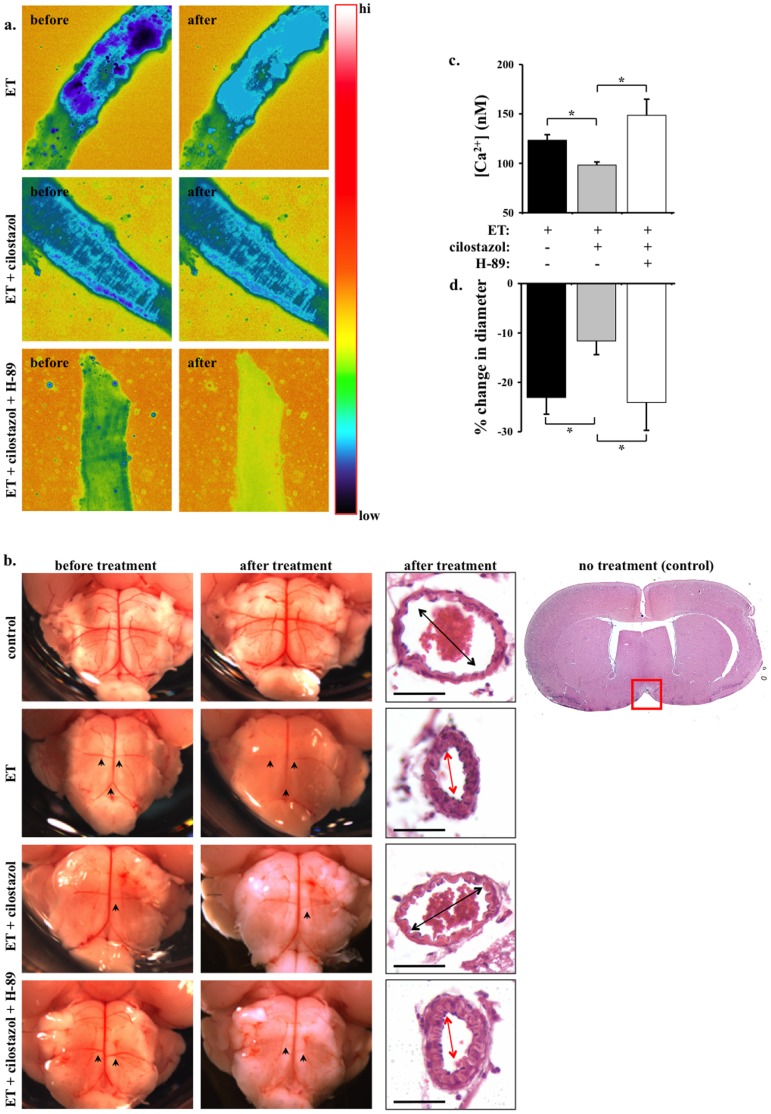
Cilostazol inhibits endothelin-induced calcium increase and vasoconstriction in mouse intracranial arteries. a. Cytosolic calcium of basilar arteries are studied *ex vivo*. Segment of artery is imaged and recorded before and after endothelin (ET) treatment in the presence or absence of cilostazol. Artery segment is pseudocolored to denote changes in cytosolic free calcium level from low to high (hi) as indicated in the color bar. **b.** Micrographs show the pons region of the whole brain before and after endothelin treatment in the presence or absence of cilostazol and/or H-89. The vascular reflection line denoting vascular tone (constriction) of intracranial arteries is adequately visible. Arrowheads point at the anterior inferior cerebellar arteries or basilar arteries to compare vascular tone before and after treatment. Sagittal sections of mouse brain are obtained at the pons, near the interpeduncular fossa boundary. A standard H&E staining is shown in the far right. Red box indicates the area of interest, where basilar artery is located. Arrows denote basilar artery constriction. Bar = 50 µm **c.** Cilostazol significantly decreases endothelin-induced cytosolic calcium increase. Administration of H-89 blocks the effect of cilostazol **d.** Changes in diameter denoting vascular constriction are expressed in percentage relative to baseline diameter prior to endothelin treatment. N = 5.

### Cell Counting

For cell counting experiments, primary smooth muscle cells were detached from femoral arteries and were grown on a 24-well plate. A total of 6 wells per group (in the present and absent of endothelin, cilostazol and/or a PKA inhibitor H-89) were used to obtain measurements. Cell numbers were measured one well every other day for about 2 weeks using hemocytometer. These experiments were repeated four times for each group (N = 4).

**Figure 4:**
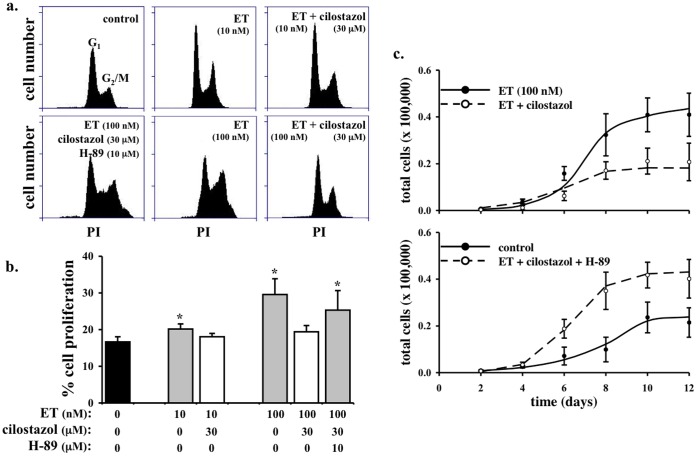
Cilostazol inhibits endothelin-induced cell proliferation in vascular smooth muscle cells. a. Endothelin (ET) at 10 and 100 nM increases cell proliferation in primary culture of smooth muscle cells as indicated by flow cytometry study. Cell proliferation is studied with nuclear marker, propodium iodide (PI). **b.** Cilostazol significantly decreases endothelin-induced cellular division. Effect of cilostazol can be blocked by H-89 (N = 3). **c.** Cell counting experiments were performed in the present and absent of ET, cilostazol and/or H-89. Cilostazol significantly decreases endothelin-induced cell growth. H-89 attenuates the effect of cilostazol in endothelin-induced cell growth (N = 4).

### Immunofluorescence Study

Fluorescence study on isolated arteries has been previously described [16], [17]. Femoral artery was isolated and cleaned from connective tissue. For basilar artery, the entire brain was used. The basic principle for this labeling technique is to optimally induce cell division followed by an optimal concentration and incubation time of 5-bromo-deoxyuridine (BrdU). In our case, cell division induced by 10 nM endothelin is optimally achieved in 24 hours *ex vivo*. Cells undergoing division tend to uptake and incorporate BrdU readily. A high concentration of BrdU or prolonged BrdU incubation would result in a high background or a possibly false positive result. Based on our titration experiments, the optimal concentration and incubation time of BrdU are determined to be 60 µM and 15 minutes, respectively. Therefore, after incubation with or without pharmacological agent(s) in DPBS for 24 hours, 60 µM of BrdU was added to the tissues for 15 minutes. The tissues were then dipped in 4% paraformaldehyde for 2 days and then embedded in 10% gelatin solution for sectioning. Once solidified, the gelatin-containing tissues were submerged in 4% paraformaldehyde for another 2 days. The artery segments or brain tissues were sectioned at 50 µm using vibratome sectioning apparatus (*Leica, Inc*.). Sample sections were then treated with 2 N HCl solution for 30 minutes and permeablized with 1% triton-X solution for 5 minutes. Anti-BrdU antibody at 1∶20 dilution for 1 hour followed with phalloidin staining at 1∶80 dilution for 1 hour were applied to the samples. The samples were then preserved with Vectashield mounting media containing DAPI and analyzed with Nikon Ti-U. Because endothelin also induces cell proliferation in other cell types, we quantified the BrdU staining in the medial part of artery, which only contains smooth muscle cells.

**Figure 5:**
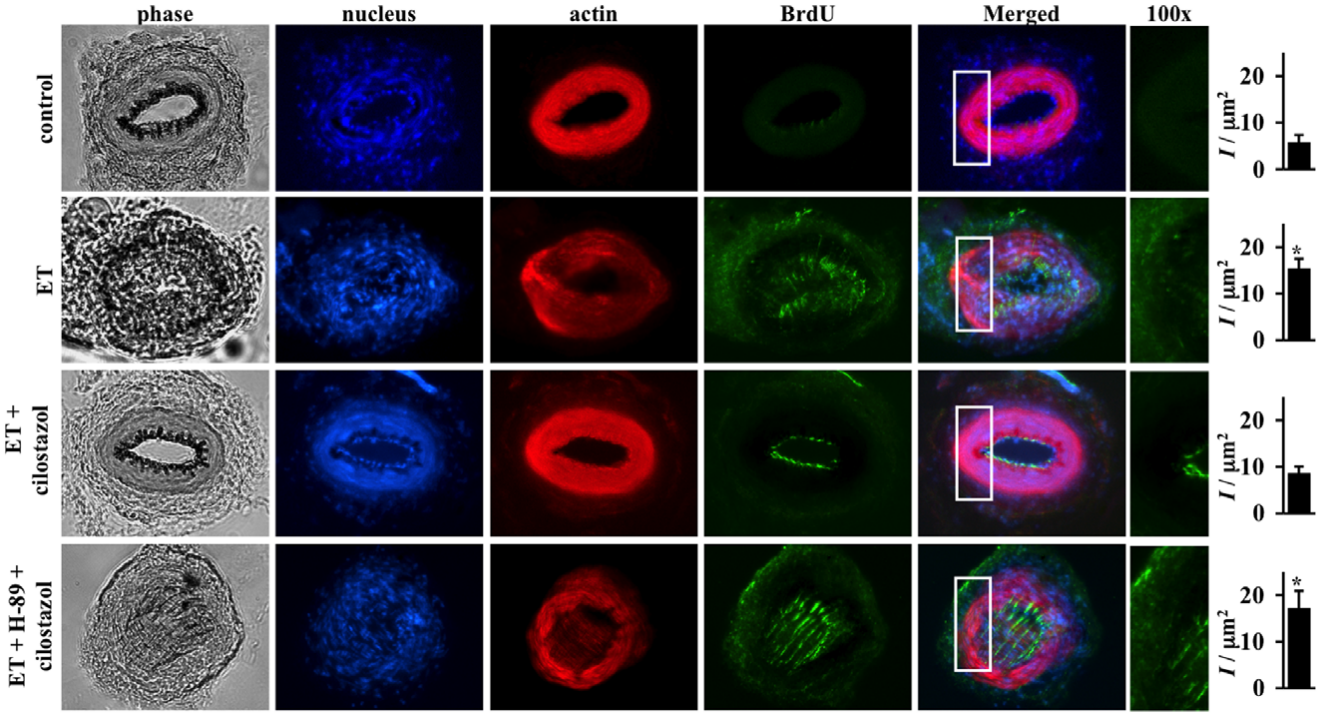
Cilostazol inhibits endothelin-induced cell proliferation in mouse femoral arteries. Effects of endothelin (ET) in the presence or absence of cilostazol and PKA inhibitor (H-89) on cell proliferation are studied with immunolabeling in femoral arteries *ex vivo*. Segment of artery is imaged and recorded for phase contrast to indicate the morphology of the artery, dapi to visualize cell nucleus, α-actin to outline contractile smooth muscle cells and BrdU to determine cellular proliferation property of the vascular cells. Endothelin increases cell proliferation as indicated by BrdU staining and cell growth into the lumen of the artery. Cilostazol blocks cell proliferation induced by endothelin, and H-89 inhibits efficacy of cilostazol. White box denotes a larger magnification. Asterisk indicates significant difference in fluorescence intensity per area within the smooth muscle cells, compared to control groups. N = 4.

**Figure 6:**
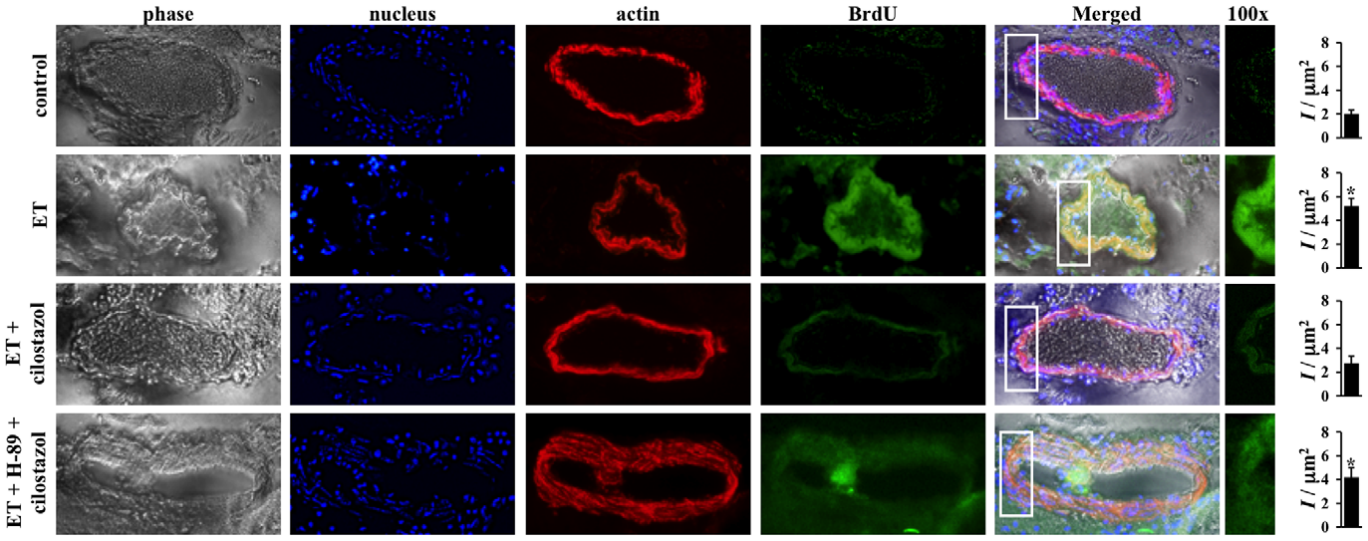
Cilostazol inhibits endothelin-induced cell proliferation in mouse basilar arteries. Effects of endothelin (ET) in the presence or absence of cilostazol and PKA inhibitor (H-89) on cell proliferation are studied with immunolabeling in basilar arteries *ex vivo*. Segment of artery is imaged and recorded for phase contrast to indicate the morphology of the artery, dapi to visualize cell nucleus, α-actin to outline contractile smooth muscle cells and BrdU to determine cellular proliferation property of the vascular cells. Endothelin increases cell proliferation as indicated by BrdU staining. Cilostazol blocks cell proliferation induced by endothelin, and H-89 inhibits efficacy of cilostazol. White box denotes a larger magnification. Asterisk indicates significant difference in fluorescence intensity per area within the smooth muscle cells, compared to control groups. N = 3.

### Immunohistochemistry Study

To better examine vasoconstriction property of basilar arteries, brains were isolated from adult mice, immediately photographed, and submerged in DPBS in the presence or absence of pharmacological agent(s). After 30 minutes incubation at 39°C, the brains were again photographed. The samples were then fixed in formalin.

Two main observations were made before and after formalin fixation. Before formalin fixation, we observed changes in the vascular reflection line. This vascular line is visible due to accumulation of red blood cells; i.e. vasodilated vasculatures contain more red cells and thus are more visible than constricted vasculatures. As such, vascular reflection line was used to indicate vascular tone of tiny arterial branches. After formalin fixation, samples were further processed for a standard procedure of H&E staining with a thickness of 5 µm. The diameter of basilar artery was more accurately measured with this type of cross-section preparation.

### Reverse-transcription Polymerase Chain Reaction (RT-PCR)

To examine distribution of PDE subtypes in smooth muscle cells, we performed semi quantitative RT-PCR studies. The primer sequences were designed based on the accession numbers obtained from the National Center for Biotechnology Information (**Table 1**). The experimental details follows previously published protocols [18], [19].

**Figure 7:**
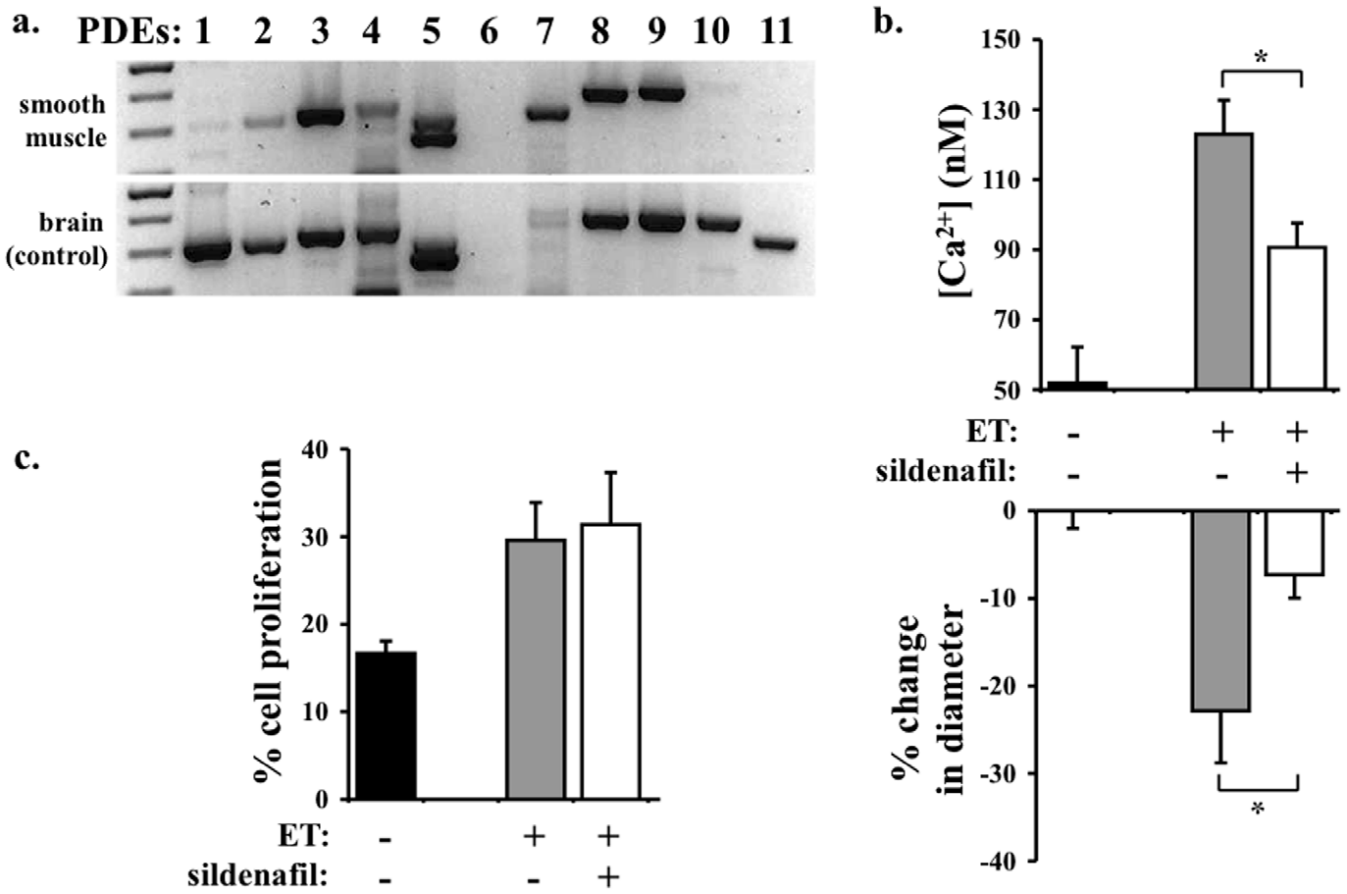
Sildenafil inhibits endothelin-induced vasoconstriction but not cellular proliferation in smooth muscle cells. a. To identify distribution of phosphodiesterase (PDE) subtypes, semi-quantitative RT-PCR was performed. Mouse brain homogenate was used as an internal control. **b.** To examine the roles of PDE5 in smooth muscle cells, we applied sildenafil on basilar arteries. Sildenafil significantly decreases endothelin-induced cytosolic calcium increase and vascular constriction. **c.** Unlike cilostazol, sildenafil does not have any effect on endothelin-induced cellular proliferation of smooth muscle cells. N = 3.

### Pharmacological Treatment

Cultured cells, artery segments or cerebral vasculatures were stimulated with 10 nM endothelin, as previously described [20], [21]. In some groups, various concentrations of cilostazol were added before or after endothelin stimulation. In other groups, cultured cells, artery segments or cerebral vasculatures were incubated in DPBS for 24 hours at 39°C with or without endothelin (10 or 100 nM) in the presence or absence of cilostazol (30 µM) and/or H-89 (10 µM). In some cases, sildenafil (30 µM) was used in the presence or absence of endothelin. In all cases, pharmacological agents were diluted to the concentrations so that the exact same volume of solution was added to the samples, in an effort to maintain identical volume. Cell and tissue culture reagents were purchased from *Cellgro, Inc.* All other reagents, including endothelin and cilostazol, were purchased from *Sigma Aldrich, Inc*, except for DAPI (*Vector Laboratories, Inc*.) and phalloidin and anti-Brdu antibodies (*Invitorgen, Inc*.).

**Figure 8:**
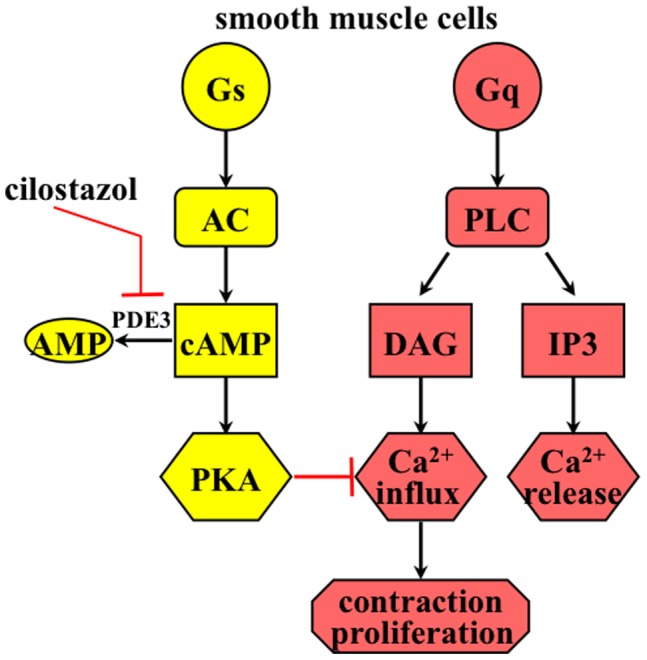
Working model depicts the roles of cilostazol on extracellular calcium influx in smooth muscle cells. Trimeric G-proteins (Gs or Gq) can activate downstream effectors, such as adenylate cyclase (AC) or phospholipase C (PLC). Endothelin receptor is coupled with Gq and can activate PLC. PLC hydrolyzes PIP2 to IP3 (induces intraorganellar calcium release) and DAG (promotes extracellular calcium influx). Cilostazol inhibits phosphodiesterase 3 (PDE3), thereby preventing degradation of cAMP to AMP. PKA (cAMP dependent protein kinase) can inhibit calcium influx in smooth muscle cells. Thus, cilostazol inhibits endothelin-induced smooth muscle contraction and cell proliferation by inhibiting calcium influx through PKA activation.

### Data Analysis

Experiments were repeated on different sets of cell populations at least three times. When primary cells were used, cells were also isolated from at least three different animals (N≥3 for each group). A minimum of six coverslips for each experiment was used for immunostaining analysis, and a minimum of five sections was cut for immunohistochemistry study. All quantifiable data were reported as mean ± SEM. Comparison between two groups was carried out using Student t-test. Data comparisons for more than two groups were done using ANOVA test, followed by Tukey posttest analysis. The difference between groups was considered statistically significant at p<0.05. All statistical analysis was done with *GraphPad Prism*, version 5.0.

## Results

### Cilostazol Specifically Blocks Extracellular Calcium Influx

Although cilostazol has been reported to alter cytosolic calcium, it is still not clear if effects of cilostazol on repressing cytosolic calcium are associated with extracellular calcium influx, intraorganellar calcium release, or by both mechanisms. In this study, we first identify the concentration of cilostazol required for an optimal response in vascular smooth muscle cells. We used the most potent vasoconstrictor (endothelin) and primary culture of smooth muscle cells isolated from the mouse femoral artery.

Endothelin at 10 nM induced a biphasic increase in intracellular free calcium, consisting of a transient peak and a subsequent sustained increase (**Figure 1a**). After removal of extracellular calcium, the transient peak was unaffected, but the sustained increase was abolished (data not shown), indicating that a sustained increase in intracellular calcium is a result from extracellular calcium influx. On the other hand, the transient increase is a result of mobilization of calcium from the intraorganellar calcium store.

The sustained increase in cytosolic calcium by endothelin was suppressed by cilostazol in a concentration-dependent manner with IC_50_ values of around 3 µM. In endothelin-induced cytosolic calcium increase, 3 µM of cilostazol is the suboptimal concentration to return cytosolic calcium to the baseline level (**Figure 1b**). We therefore used this concentration throughout our experiments with cilostazol. To differentiate effects of cilostazol on calcium influx versus calcium release, we pre-incubated the cells with cilostazol (**Figure 1c**). In the presence of cilostazol, endothelin has no effect in maintaining a steady increase in cytosolic calcium, indicating that cilostazol selectively blocks calcium influx. To confirm this finding, we examined the effect of cilostazol on 10 or 100 µM thapsigargin-induced calcium release (**Figure 1d or 1e**). Consistent with our previous results, cilostazol has minimal or insignificant effect on intraorganellar calcium release.

### Cilostazol Inhibits Intracelluar Calcium Increase and Vasoconstriction in Mouse Femoral Arteries through PKA

To further investigate the inhibitory effect of cilostazol on extracellular calcium influx and vasoconstriction, we challenged femoral arteries with endothelin *ex vivo* (**Figure 2a**). Endothelin not only increases cytosolic calcium but also induces vasoconstriction of blood vessel. Submaximal concentration of cilostazol (30 µM) inhibits cytosolic calcium increase and vasoconstriction induced by 10 nM endothelin. These data suggest that inhibiting calcium influx by cilostazol is sufficient to prevent endothelin-induced vasospasm.

Because cilostazol inhibits phosphodiesterase-3 (PDE3), increases cAMP level and thereby activates cAMP-dependent protein kinase (PKA), we used PKA inhibitor (H-89) to identify a potential molecular mechanism in the inhibiting effects of cilostazol in our experimental model. Our data show that H-89 could block efficacy of cilostazol-induced calcium influx (**Figure 2b**) and vasoconstriction (**Figure 2c**).

### Cilostazol Inhibits Intracelluar Calcium Increase and Vasoconstriction in Intracranial Arteries through PKA

Because the most prominent pathological relevance of endothelin-induced cellular proliferation and vasospasms occurs during stroke, we extended our studies using brain tissues. Due to technical challenges with intracranial arteries, our calcium and constriction studies were performed separately. As expected, endothelin increases cytosolic calcium in basilar arteries (**Figure 3a**). Submaximal concentration of 30 µM cilostazol inhibits endothelin-induced cytosolic calcium increase.

To examine effects of cilostazol on the brain vasculature, intracranial arteries were compared before and after cilostazol treatment in the presence and absence of endothelin and/or H-89 (**Figure 3b**). Because of the minute sizes of the cerebral arteries in the mouse, we used vascular reflection line as a measurement of vascular tone. Of note is that endothelin has a greater vasoconstriction effect on smaller rather than larger arterial branches. Thus, the effects of cilostazol on endothelin-induced constriction are more noticeable in anterior inferior cerebellar arteries and basilar arteries. To further examine the anatomy of basilar arteries more closely, we analyzed sagittal sections at the area of pons-interpeduncular fossa boundary. Consistent with our femoral studies, endothelin can cause intracellular calcium increase (**Figure 3c**) and vasoconstriction (**Figure 3d**). Furthermore, H-89 can block the inhibitory effects of cilostazol.

### Cilostazol Inhibits Cell Proliferation in Vascular Smooth Muscle Cells through PKA

While the short-term effect of endothelin is to induce vasoconstriction, the long-term effect of endothelin is known to promote mitogenic response (cellular proliferation) [12], [13]. To examine if cilostazol can also inhibit the long-term effect of endothelin, we analyzed endothelin-induced cell proliferation in isolated smooth muscle cells in the presence and absence of cilostazol and/or H-89 (**Figure 4a**). Endothelin induces cell proliferation in a dose-dependent manner (10 vs. 100 nM; **Figure 4b**). The effect of endothelin on cell proliferation is inhibited by cilostazol, as indicated by nuclear marker propidium iodide. In colorimetric cell proliferation assay, cilostazol also inhibits cell proliferation induced by 10 nM endothelin in a concentration-dependent manner with IC_50_ values of around 3 µM (data not shown). Our *in vitro* studies were further confirmed by cell counting experiments (**Figure 4c**). Endothelin induces increase in cell numbers, which can be repressed by cilostazol. Furthermore, the effect of cilostazol on cell growth can be blocked with H-89.

### Cilostazol Inhibits Cell Proliferation in Femoral and Basilar Arteries through PKA

To verify the potential inhibitory mechanism on cell proliferation from our *in vitro* data, we next investigated the effects on cilostazol *ex vivo* using isolated mouse femoral and basilar arteries. We used BrdU as a marker of cell proliferation. In both femoral (**Figure 5**) and basilar (**Figure 6**) arteries, endothelin induces a gross cell proliferation as indicated by both dapi and BrdU florescence images. The effect of endothelin in cell growth is inhibited with cilostazol treatment. As expected, inhibiting PKA blocks efficacy of cilostazol on cell growth. Of note is that simultaneous administration of endothelin and H-89 also causes intima thickening, as a result from the peripheral cell proliferation. Our studies demonstrate that cilostazol can inhibit cell growth induced by endothelin in both peripheral and intracranial arteries.

### Sildenafil Inhibits Endothelin-induced Vasoconstriction but not Cellular Proliferation in Smooth Muscle Cells

To examine the specificity of cilostazol effects on its vasoconstriction and proliferation inhibitions, we screened the distribution of PDE subtypes in smooth muscle cells. As expected, PDE3 is clearly present in the smooth muscle cells (**Figure 7a**). Because other PDE subtypes are also detected, we chose to identify effects of PDE5 inhibition. Sildenafil, a specific PDE5 inhibitor, blocks endothelin-induced cytosolic calcium increase and basilar artery contraction (**Figure 7b**). Unlike cilostazol, however, sildenafil has no effect on cellular proliferation of smooth muscle cells (**Figure 7c**). All in all, our results indicate that cilostazol can specifically inhibit vasoconstriction and cell growth induced by endothelin in vascular smooth muscle cells.

## Discussion

In the present study, we show for the first time that cilostazol has a unique property to block activity of endothelin-induced vasospasm and cellular proliferation (**Figure 8**). Our *in vitro* and *ex vivo* studies further demonstrate that cilostazol blocks endothelin function by inhibiting endothelin-induced extracellular calcium influx. Endothelin promotes cellular proliferation phenotype in vascular smooth muscle cells and mouse femoral and basilar arteries. Furthermore, the action of cilostazol on blocking this cellular proliferation depends on the activity of PKA. Our studies suggest a possible mechanism of cilostazol in treating patients associated with endothelin overload, such as hyperproliferation and vasospasm.

Cilostazol is a phosphodiesterase inhibitor that prevents platelet aggregation by increasing cAMP level and thereby activating PKA. Although cilostazol is FDA-approved agent for treating intermittent claudication, it has been suggested that cilostazol can treat other cardiovascular diseases. Based on its vasodilation activity, for example, cilostazol has been proposed to be a possible regimen for patients with congestive heart failure, ischemic stroke, post-stent thrombosis, and other atherothrombotic vascular diseases [1], [2]. Based on its anti-proliferation activity, cilostazol has also been suggested to reduce cardiac hypertrophy and attenuate the overall cardiac remodeling [22]. In the present study, we examined the effects of cilostazol in the presence of endothelin, which is known to be a potent vasoconstrictor and cell growth promoter.

Endothelin is known to be the most potent endogenous growth promoting and vasoactive peptide. It is therefore not surprising that over production of endothelin has been associated with many types of cancer and cardiovascular disorders. Accordingly, endothelin receptor blockers (such as atrasentan and bosentan) have been clinically tested in ovarian cancer and pulmonary hypertension [13], [23], [24]. More importantly, one of the endothelin blockers (clazosentan) is currently under phase-3 clinical trial to treat patients with aneurysmal subarachnoid hemorrhage [25]. The consensus is that endothelin plays a major role in acute vascular injury. In particular, endothelin contributes to major complications resulting in morbidity and mortality of patients with stroke [7]–[9].

We and others have previously shown that endothelin can induce both extracellular calcium influx and intraorganellar calcium release in smooth muscle cells [12], [13]. In the present study, we show that while cilostazol has a minor effect on endothelin-induced calcium release, it has a significant inhibitory effect on calcium influx in a dose-dependent manner. We further show that pre-treatment of cilostazol also has equipotent efficacy on calcium influx but not on calcium release. To further confirm the mechanism of action of cilostazol, we utilized thapsigargin to induce intracellular calcium release. As expected, we consistently observe that cilostazol has a minor role on calcium release, induced by either endothelin or thapsigargin. To validate our *in vitro* results, we performed *ex vivo* studies on mouse femoral arteries. As expected, endothelin causes an increase in cytosolic calcium and vasoconstriction. These effects are blocked in arteries after 30 minutes pre-treatment with cilostazol. Cilostazol also shows a similar effect on arterial segments challenged with endothelin first (not shown). Regardless, our studies on cultured cells *in vitro* and isolated tissues *ex vivo* show that cilostazol has an inhibitory effect on calcium influx and vasoconstriction.

We and others have also shown that endothelin can induce cellular proliferation in vascular smooth muscle cells [20], [26]. Furthermore, it has been shown that calcium influx can promote cellular proliferation [27]. To examine effect of cilostazol on proliferation by endothelin-induced calcium influx, we performed a standard cellular proliferation assay. Consistent with previous studies [20], [26], endothelin significantly increases baseline level of cellular proliferation. Pre-treatment with cilostazol is sufficient to block cellular proliferation induced by endothelin in primary cultured smooth muscle cells. To confirm these *in vitro* results, we performed *ex vivo* studies on mouse femoral and basilar arteries. As indicated by nuclear marker (dapi), endothelin increases cell numbers, where cells have grown toward the lumen of the artery segment. As indicated by proliferative marker (BrdU), endothelin increases cellular proliferation, especially on the newly dividing cells around the lumen of the artery.

It has been known for a while that endothelin plays an important role in hypertension and stroke. However, the impact of endothelin in cerebral microcirculation is not known. In particular, a high level of circulating endothelin is detected in patients with stroke [7]–[9]. It has been hypothesized that endothelin plays a major role in vascular injury and contributes to seizures, coma and death in these patients. We thus examine the effects of endothelin and cilostazol in the intracranial arteries. Consistent with our cell culture *in vitro* and femoral artery *ex vivo* studies, cilostazol also inhibits endothelin-induced vasoconstriction in basilar artery *ex vivo*. The vasoconstriction effect of endothelin is especially more apparent in the anterior inferior cerebellar arteries. Endothelin-induced vasoconstriction is further confirmed in basilar arteries and other intracranial arteries surrounding Circle of Willis (not shown). Most important is that cilostazol can sufficiently inhibit the effect of endothelin.

Because inhibition of PDE3 has been shown to increase PKA activity, we used PKA inhibitor (H-89) to examine the molecular mechanism of cilostazol. Our data suggest that PKA inhibition can abolish the inhibitory effect of cilostazol on endothelin. It is generally known that PKA can block both calcium influx [28], [29] and cellular proliferation [30]. Furthermore, cilostazol has been shown to increase PKA activity through an increase in baseline cAMP level [6], [31]. We therefore propose that inhibitory effects of cilostazol on endothelin depend on both calcium influx and/or PKA activity.

Supporting our working model (**Figure 7**), we used a specific PDE5 inhibitor sildenafil in our experimental system. Although sildenafil blocks endothelin-induced cytosolic calcium increase and basilar artery contraction, it has no effect on cellular proliferation of smooth muscle cells. PDE5 has been shown to promote cGMP degradation. This is consistent with our previous work demonstrating that cGMP decreases cytosolic calcium and induces vasodilation [32]–[34]. In summary, our studies provide the first evidence on the specific-dual inhibitory effects of cilostazol on endothelin-induced vasoconstriction and cellular proliferation. Our studies also offer a possible mechanism by which cilostazol has been tried in clinical study associated with stroke [1]. Together, we propose that cilostazol has a therapeutic benefit to post-stroke patients where endothelin has been shown to complicate the progression of the recovery.
